# Determinants of viral load suppression among adolescents on antiretroviral therapy in Eswatini: a cross-sectional study

**DOI:** 10.1186/s12879-025-10872-z

**Published:** 2025-04-10

**Authors:** Londiwe D. Hlophe, Constance S. Shumba, Diribsa T. Bedada, Peter S. Nyasulu

**Affiliations:** 1 Division of Epidemiology and Biostatistics, Faculty of Medicine and Health Sciences, Stellenbosch University, Cape Town, South Africa; 2 Division of Epidemiology and Social Sciences, Institute for Health and Equity, Medical College of Wisconsin, Milwaukee, WI United States of America; 3 Department of Public Health, College of Health Sciences, National Defence University, Addis Ababa, Ethiopia; 4 Division of Epidemiology and Biostatistics, School of Public Health, Faculty of Health Sciences, University of the Witwatersrand, Johannesburg, South Africa

**Keywords:** HIV, Viral load suppression, Adolescents living with HIV, Kingdom of eswatini, Antiretroviral therapy

## Abstract

**Background:**

The goal of antiretroviral therapy (ART) is to achieve a sustained HIV suppressed viral load. However, adolescents often present poor adherence to ART which is associated with lower rates of viral load suppression (VLS). The objective of this study was to determine the viral load suppression levels and the associated factors among adolescents living with HIV (ALHIV) and on ART in Eswatini.

**Methods:**

We conducted cross-sectional analysis of data from 911 adolescents aged 10 to 19 living with HIV and on ART between the period January 2017 and September 2022. We collected data of demographic and clinical variables, using a standardized data abstraction tool. We defined viral load suppression as the latest viral load result of ≤ 1000 copies/ml. Univariable and bivariable logistic regression analysis was done to identify factors associated with VLS and factors with *p* < 0.1 were included in the multivariable regression analysis to adjust for the confounding effect of other variables such as age, sex, and duration of ART. Factors with *p* < 0.05 were considered statistically significant.

**Results:**

Among the 911 participants, 60% (457) were female. The mean age of the participants was 16.3 years, with mean duration on ART of 1.8 years. Viral suppression was attained by 88.5% (806/911) of the participants. Residence in the Shiselweni region was an independent factor associated with viral load suppression (aOR 0.37; 95% CI 0.15–0.19; p˂0.027).

**Conclusion:**

Low VLS is a risk factor for increased viral resistance and perpetuates HIV transmission within the population. Achieving viral suppression among ALHIV in Eswatini is challenging as data shows that VLS is way below the UNAIDS 95% cut off level among individuals on ART. This is particularly more problematic in the Shiselweni region, where viral suppression is lower than the other regions. Therefore, reinforcement of public health interventions is needed to improve treatment support for achieving sustained viral suppression among ALHIV in Eswatini.

**Supplementary Information:**

The online version contains supplementary material available at 10.1186/s12879-025-10872-z.

## Introduction

Globally, remarkable strides have been achieved in the management of Human Immuno-deficiency Virus (HIV), resulting in commendable achievements in viral load suppression (VLS) among people living with HIV (PLHIV) [1]. VLS is defined as viral load ≤ 1000 RNA copies per ml of blood and is critical in prevention of transmission of HIV to sero-negative partner, disease progression, opportunistic diseases, and AIDS-related deaths [2, 3]. Despite high rates of ART uptake and VLS observed in the general population, adolescents consistently report lower rates, despite comprising approximately 5% of all PLHIV [1, 4]. This discrepancy is further exacerbated among adolescents living with HIV (ALHIV) in sub-Saharan Africa (SSA), where VLS rates are notably lower compared to other regions [1, 4]. In 2021, VLS was 30% among the 43% ALHIV on ART in SSA [5]. Furthermore, a systematic review including studies conducted between 2010 and 2022, reported a VLS rate of 55% among ALHIV in SSA [6].

The Kingdom of Eswatini, formerly known as Swaziland, has the highest HIV prevalence of 27% globally with an incidence of 1.4 among those 15 years and above [7]. Females are disproportionately affected in the country with an HIV prevalence of 32.5% compared to 20.4% among males [8]. Additionally, the HIV prevalence is notably high among female adolescents aged 15 to 19 years compared to their male counterparts, with rates of 7.3% versus 3.9% respectively [8]. The urgency of HIV prevention and management is critical among adolescents not only in Eswatini, but in the sub-Saharan Africa (SSA) region which is home to 90% of adolescents living with HIV (ALHIV) [4].

Eswatini is among the first countries globally to meet the 95-95-95 (95% people living with HIV knowing their HIV status, 95% of these on ART and 95% of those on ART viral load suppressed) UNAIDS global targets in the general population ahead of the year 2025 [9]. Viral load suppression (VLS) was reported to be 96% as of 2022 in the country, marking a significant increase from 91% in 2017 [8, 10]. This remarkable achievement has been attributed to the uninterrupted access and availability of ART among those testing positive to HIV and programmes aimed at supporting ART adherence [10].

However, these positive gains are only observed among the general population yet when centring on only adolescents and young people living with HIV, they still present poor HIV treatment outcomes [11]. In 2017, while Eswatini reported 89% of the PLHIV on ART with 91% of them virally suppressed, among ALHIV and young people, only 81.7% were on ART and 76.4% virally suppressed [8]. The discrepancies in VLS rate among adolescents and general population, are an indication of poor adolescents’ specific HIV prevention and management strategies, and sub-optimum ART adherence among ALHIV [12, 13, 14, 15]. Studies have reported multiple barriers to ART adherence among ALHIV. These barriers include stigma and discrimination, fear of unintended disclosure associated with lack of privacy and medication packaging, lack of support from family, school and community, competing time for school, personal and medication related activities, side effects of medication and facility based factors such as distance to health facility and attitudes of health care workers [16, 17, 18, 19]. However, in Eswatini, adolescent specific data are limited due to the long description age band of 15 to 24 years where adolescents are reported with young people and paediatrics. This study thus aimed to assess the VLS and associated factors among ALHIV aged 10 to 19 years on ART in Eswatini.

## Methods

### Study design

We conducted a cross-sectional study using routinely collected records of adolescent on ART in Eswatini between January 2017 and September 2022. Data were extracted from the Client Management Information System (CMIS).

Our interest in data from 2017 stems from Eswatini adoption and implementation of the universal test and treat (UTT) guidelines during that year, coinciding with the rollout of the CMIS in all the health facilities nationwide [20, 21]. The CMIS serves as a national electronic medical record system designed to monitor and provide care for individuals accessing healthcare facilities across the country. The system consolidates all the medical history of the individuals into one record allowing for comprehensive health information management [20].

### Inclusion and exclusion criteria

This study included records of ALHIV who were initiated on ART at age 10–19 years with either vertical or horizontal HIV infection. Only records of ALHIV who had been on ART for a minimum of 6 months, in accordance with Eswatini HIV management guidelines for viral load testing, were included in the analysis [21]. ALHIV records with missing key variables such as age, viral load result and ART initiation date were excluded from the analysis.

### Study setting

As of 2021, there were 10,153 adolescents living with HIV (ALHIV) in Eswatini accessing care through various differentiated ART service delivery models [22]. These models include multi-month (3 and 6 months) refills, mainstream, fast track, as well as expert client approach offered by lay heath workers (LHWs) through Teen Clubs [21, 23].

Multi-month is a model of ART care in which stable adolescents receive their ART refill every three or six months. This model caters for individuals who are mobile such as those studying outside the borders of the country. Clinical screening is done during the refill date which is either at three or six months [21]. Meanwhile, the mainstream model, also known as the standard of care model provides monthly direct patient monitoring by a nurse or a doctor mostly for patients newly initiated in ART, with detectable viral load, with ART adherence issues, or suspected treatment failure [21]. The fast track is another ART care model which is designed to reduce the time spent at the health facility by allowing stable individuals to pick up their medication straight from the pharmacy [24]. Routine clinical screenings for individuals enrolled in the fast-track model are scheduled separately from their refill dates, which occur every three or six months [21]. Lastly, Teen Clubs in which adolescents are afforded support through peer led sessions facilitated by LHW [21]. In 2021, there were 81 Teen Clubs catering for 3742 adolescents aged 10 to 19 years as members [22]. Teen Clubs cater to both stable adolescents and those with detectable viral loads, offering monthly sessions with refill services provided by healthcare workers, including nurses and doctors [24]. The expert client approach involves training LHWs on HIV treatment adherence, stigma and disclosure, HIV linkage to care, and communication and counselling skills [25, 26].

The country health infrastructure comprised of 327 health facilities, including one (1) national referral hospital, three (3) specialized hospitals, five (5) regional referral hospitals, five (5) health centres, six (6) public health units, 297 clinics of which 65 are specialized clinics, and nine (9) private hospitals [27]. These health facilities include public institutions characterized by free or subsidized medical services, such as those owned by the government, non-profit organizations, and missionary organizations. In contrast, private health facilities operate on a for-profit basis [27]. These health facilities are distributed in the four regions (Hhohho, Manzini, Lubombo and Shiselweni) of the country with the national referral hospital in the Hhohho region [28].

### Study sample

A total of 3,420 ALHIV aged 10 to 19 years who were initiated on ART were identified. Using the OpenEpi sample calculator [29] to determine the required number of participants, a minimum sample size of 260 records was necessary. This calculation was based on a 95% confidence level, 5% margin of error, and an estimated 76% viral load suppression rate derived from the 2017 national rates among adolescents and young adults [8].

**Fig. 1:**
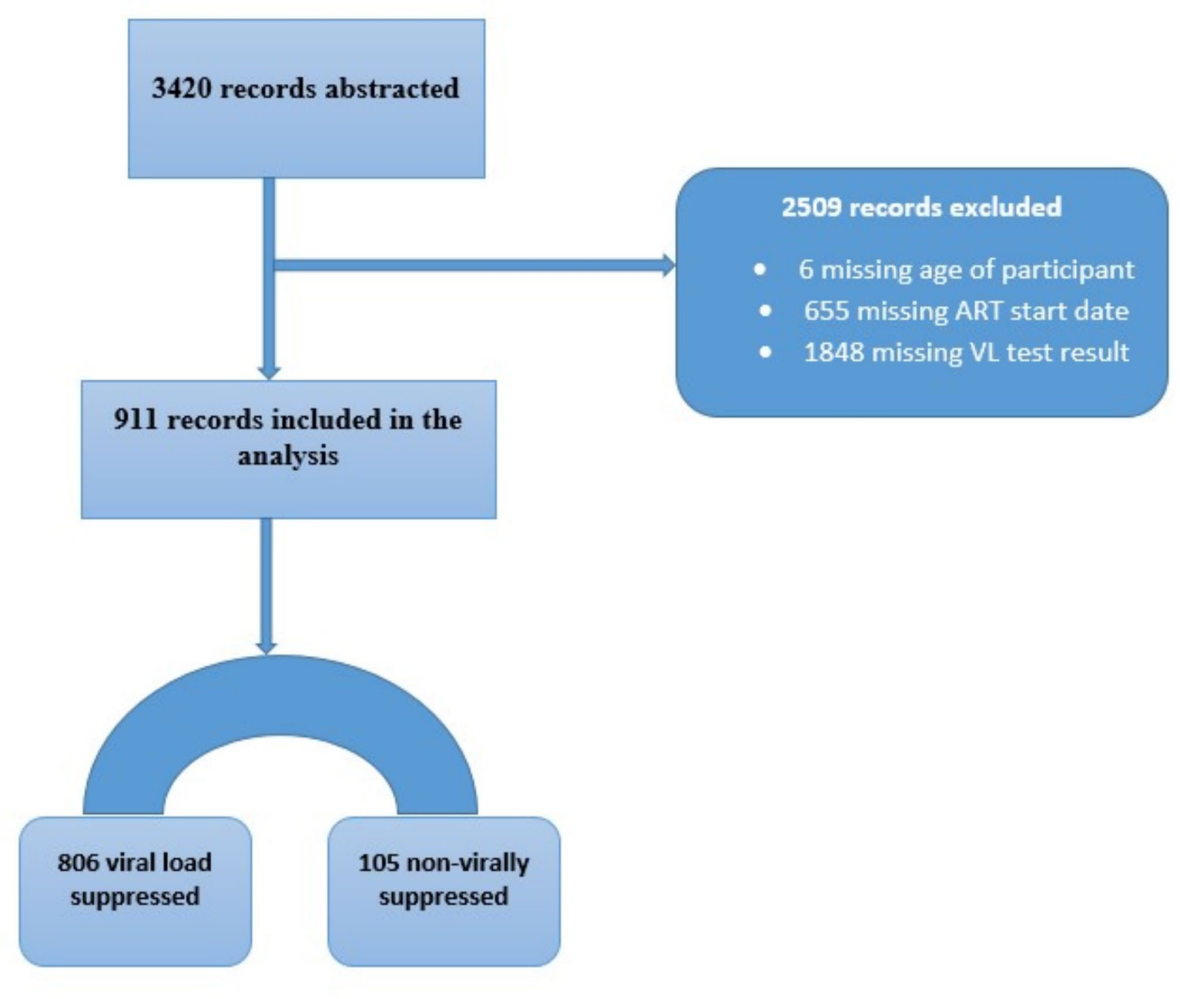
Flow diagram illustrating total number of records included in analysis from the abstracted records

Despite this, all available records were considered. Of the total records reviewed, 911 (26.6%) met the inclusion criteria and were included in the analysis (Fig. 1). The remaining 2,509 (73.4%) records were excluded due to missing key characteristics such as viral load results and ART initiation dates. A significant proportion of individuals with missing data, such as viral load results and ART initiation dates, were from the Manzini region (1,008). The distribution of missing key variables such as viral load results across other regions was as follows: Hhohho (536), Lubombo (573), and Shiselweni (392) (Supplementary file).

The missingness of key variables, particularly viral load results, was notably high during the COVID-19 pandemic in 2020 and 2021, with 1,217 records lacking viral load data. By contrast, post-COVID-19 in 2022, only 231 records were missing viral load results, while in 2017, 109 records were missing this data.

The following variables had incomplete data due to missingness: “ART regimen (464/911) and model of ART care (57/911)”. The missingness pattern was assessed by modelling the probability of missingness as a function of observed variables. The results indicated that the probability of missingness was dependent on only observed information, consistent with a Missing at Random (MAR) mechanism. Multivariate Imputation by Chained Equations (MICE) was used to impute missing data of these variables prior to regression analysis. A sensitivity analysis comparing the results from the complete-case analysis and the imputed datasets showed that the overall conclusions remain consistent, with only slight differences in regression coefficients.

### Study variables and measurements

The primary objective of this study was the assessment of viral load suppression, which was defined as having a viral load equal to or less than 1000 RNA copies per millilitre of blood. This criterion aligns with the standard viral load suppression measurement utilized in Eswatini [21]. For consistency and accuracy, participant’s most recent viral load result taken within six months before data abstraction was utilized in the analysis thus a single RNA measurement was used to define VLS.

In Eswatini, RNA testing is conducted after 6 months of ART initiation which is then repeated at 12 months (6 months after the first one). After two consecutive undetectable viral load, RNA testing is conducted yearly [24].

In addition to viral load suppression, various demographic, social, HIV-related, and facility-related variables were considered. Demographic and social factors encompassed variables such as sex, age, and region of residence. Key HIV-related variables included age at diagnosis, ART start date, current model of ART care, ART regimen, and CD4 count results.

CD4 count was determined as the most recent measurement taken prior to data abstraction. CD4 count results were further categorized according to the WHO clinical staging system (stage I-II and stage III-IV), where stage I-II is defined as CD4 + cell count ≥ 200) and stage II-IV is CD4 + cell count < 200) [21, 30]. ART regimen was classified into two main categories: first-line (comprising a combination of two nucleoside reverse-transcriptase inhibitors (NRTIs) and one non-nucleoside reverse transcriptase inhibitor (NNRTI)) or 2NRTIs plus 1 integrate inhibitor (INSTI)) and second-line (comprising a combination of two NRTIs and one ritonavir-boosted protease inhibitor (PI)), following the Swaziland integrated HIV management guidelines [21]. Also, current model of ART care was categorized into four distinct approaches based on how ART refill services are provided to adolescents in Eswatini. These approaches are multi-month, mainstream, fast track, and Teen Clubs [23].

Lastly, facility ownership was also considered, with categorizations including government-owned, non-governmental organization (NGO)-owned, privately owned, and mission-owned facilities.

### Data management

The data were initially received in a Microsoft Excel format and subsequently imported into Stata 16 for further processing and analysis. In Stata, variables were meticulously constructed and defined in accordance with the specified measurements and parameters of the study.

To ensure the integrity and reliability of the dataset, a rigorous data cleaning process was undertaken. This process commenced with the execution of descriptive statistical analyses to identify any anomalies, errors, inconsistencies, or missing values within the dataset. Missing data points were uniformly coded as “999” for clarity and ease of identification.

Observations with missing data points pertaining to the outcome variable were systematically excluded from subsequent analyses. This step was undertaken to maintain the robustness and validity of the analytical procedures employed.

### Data analysis

Data were analyzed using Stata statistical software, release 16 [31]. Descriptive analysis was conducted, wherein continuous variables such as age, age at HIV diagnosis, and years on ART were presented as means with standard deviations. Categorical variables were summarized as frequencies and percentages. Bivariable analysis was conducted using the chi-square test for categorical variables. Factors that were significant at *p* ≤ 0.1 were further evaluated in the multivariable logistic regression model, with age and sex included a priori in the multivariable analysis. All variables with a *p*-value ≤ 0.05 were considered statistically significant and retained in the final model.

## Results

### Characteristics of participants

The mean age of ALHIV and on ART was 16.3 years (SD = 3.3). Out of the total 911 participants, 606 (66.5%) were aged between 15 and 19 years, with 155 (60%) of them being females. The Hhohho region accounted for the highest proportion of adolescents, totalling 355 (39%), while the Shiselweni region had the lowest representation, with only 133 (15%) adolescents on ART (Table 1).

Of the 911 participants, only 325 (35.7%) were diagnosed with HIV between 2017 and 2022, while 586 were diagnosed before 2017 and initiated ART care between 2017 and 2022. Among those diagnosed from 2017 onwards, the mean age at HIV diagnosis was 11.8 years (SD 3.99) while mean age at ART initiation was 12 years of age (SD 3.5). On average, ALHIV spent 7 month (SD 2 years) between diagnosis and ART initiation and had been on ART for at least 1.8 years (SD 0.37).

Overall, VLS among adolescents was 88.5%. Within the different age groups, those aged 15 to 19 years had the highest VLS, which was 89.3%, while those aged 10 to 14 years had VLS of 86.9%. Regionally the distribution of VLS was as follows: the Hhohho region 90.7%, Manzini 89.5%, Lubombo 87.5%, and Shiselweni 81.95% (Table 1).

**Table 1:** Characteristics of study participants stratified by VLS (*N* = 911)

Variable	Total *n*/*N* (%) ^a^	VLS (*n*, %)	Non-viral suppressed (*n*, %)	*p*-value
**Age group**				
10–14	305/911 (33.5)	265 (86.89)	40 (13.11)	0.287
15–19	606/911 (66.5)	541 (89.27)	65 (10.73)	
**Sex**				
Female	551/911 (60.5)	490 (88.93)	61 (11.07)	0.595
Male	360/911 (39.5)	316 (87.78)	44 (12.22)	
**Region**				
Hhohho	355/911 (39.0)	322 (90.70)	33 (9.30)	0.052
Lubombo	184/911 (20.2)	161 (87.50)	23 (12.50)	
Manzini	239/911 (26.2)	214 (89.54)	25 (10.46)	
Shiselweni	133/911 (14.6)	109 (81.95)	24 (18.05)	
**Facility Ownership**				
Government	641/911 (70.4)	567 (88.46)	74 (11.54)	0.945
NGO	123/911 (13.5)	108 (87.80)	15 (12.20)	
Faith-based	147/911 (16.1)	131 (89.12)	16 (10.88)	
**ART Duration**				
< 2 years	153/911 (16.8)	139 (90.85)	14 (9.15)	0.313
≥ 2years	758/911 (83.2)	667 (87.99)	91 (12.01)	
**ART Regimen**				
1st Line	430/447 (96.2)	387 (90)	43 (10)	0.074
2nd Line	17/447 (3.8)	13 (76.5)	4 (23.5)	
**WHO Clinical Stage**				
I-II	81/141 (57.4)	72 (88.89)	9 (11.11)	0.143
III-IV	60/141 (42.6)	40 (80)	12 (20)	
**Model of ART Care**				
Multi-Months	66/854 (7.7)	64 (96.97)	2 (3.03)	0.058
Mainstream	433/854 (50.7)	379 (87.53)	54 (12.47)	
Teen Clubs	338/854 (39.6)	302 (89.35)	36 (10.65)	
Fast Track	17/854 (2.0)	17 (100)	0 (0)	

^a^ some sample sizes for variables may not add up to *N* = 911 due to missing data

### Factors associated with viral load suppression among adolescents living with HIV

In the bivariable logistic regression analysis, both residency in the Shiselweni region (OR 0.47, 95% CI: 0.47–0.82) and receiving ART care under the mainstream model at the current treatment facility (OR 0.22, 95% CI: 0.05–0.92) were statistically significant predictors of viral load suppression (VLS) and were subsequently included in the multivariable logistic regression model (Table 2).

In the multivariable regression model, residency in the Shiselweni region (aOR = 0.37, 95% CI: 0.15–0.91) was a significant factor associated with VLS (Table 2). This suggests that adolescents residing in the Shiselweni region were less likely to achieve viral load suppression compared to those in other regions, even after adjusting for other factors in the model.

**Table 2:** Factors associated with VLS among ALHIV

		Bivariable analysis		Multivariable analysis (Complete case analysis)		Multivariable analysis (MICE)	
Variable	Total n/N (%)	OR (95% CI)	*P*-value	aOR (95%CI)	*P*-value	aOR (95%CI)	*P*-value
**Age**							
10–14	305/911 (33.5)	Ref		Ref		Ref	
15–19	606/911 (66.5)	1.26 (0.83–1.91)	0.287	1.28 (0.72–2.21)	0.347	1.49 (0.81–2.54)	0.231
**Sex**							
Female	551/911 (60.5)	Ref		Ref		Ref	
Male	360/911 (39.5)	0.89 (0.59–1.35)	0.595	1.06 (0.64–1.73)	0.833	1.35 (0.72–1.98)	0.632
**Region**							
Hhohho	355/911 (39.0)	Ref		Ref		Ref	
Lubombo	184/911 (20.2)	0.72 (0.41–1.26)	0.249	0.78 (0.4–1.55)	0.481	0.54 (0.23–1.28)	0.302
Manzini	239/911 (26.2)	0.88 (0.51–1.52)	0.639	0.88 (0.48–1.63)	0.686	0.89 (0.35–2.11)	0.776
Shiselweni	133/911 (14.6)	0.47 (0.26–0.82)	0.008^*^	0.48 (0.24–0.94)	0.036^*^	0.37 (0.15–0.91)	0.027*
**Facility Ownership**							
Government	641/911 (70.4)	Ref					
NGOs	123/911 (13.5)	0.92 (0.51–1.67)	0.789				
Faith-based	147/911 (16.1)	1.07 (0.60–1.89)	0.820				
**ART Duration**							
< 2 years	153/911 (16.8)	Ref					
≥ 2 years	758/911 (83.2)	0.74 (0.41–1.33)	0.315				
**ART Regimen**							
1st Line	430/447 (96.2)	Ref		Ref		Ref	
2nd Line	17/447 (3.8)	0.36 (0.11–1.16)	0.086	0.05 (0.00-1.30)	0.072	0.03 (0.00-1.07)	0.054
**WHO staging**							
I-II	81/141 (57.4)	Ref					
III-IV	60/141 (42.6)	2.00 (0.78–5.11)	0.148				
**Current ART Model of Care**							
Multi-Month	66/854 (7.7)	Ref		Ref		Ref	
Mainstream	433/854 (50.7)	0.22 (0.05–0.92)	0.038*	0.96 (0.48–1.91)	0.903	1.23 (0.67–2.23)	0.661
Teen Clubs	338/854 (39.6)	0.26 (0.06–1.12)	0.070	0.90 (0.47–1.7)	0.739	0.94 (0.58–2.14)	0.623

^*^Statistically significant

## Discussion

The study revealed that overall, viral load suppression (VLS) among Swati ALHIV aged 10 to 19 was 88.5%, which falls below both the national average of 96% among all people living with HIV and on ART and the UNAIDS target of 95% VLS among individuals on ART [10, 32]. While the VLS among Swati ALHIV is comparable to VLS reported in other studies conducted in sub-Saharan African countries such as Uganda (81%), and Kenya (85.6%), it is significantly higher than findings from studies conducted in other countries within the region, such as Ethiopia (73.1%) and South Africa (64%) [13, 33, 34, 35]. The differences observed in VLS among these countries may be due to variations in outcome measurements, study settings, sample sizes, study designs among others [36]. However, it is essential to note that an unsuppressed viral load is linked to opportunistic infections, drug resistance, and ultimately, increased mortality [3]. Therefore, the reported viral load suppression underscores the importance of enhanced interventions to address barriers to viral suppression in specific contexts. Efforts aimed at strengthening healthcare systems to improve adherence to ART are needed to achieve optimal treatment outcomes and reduce the burden of HIV in the adolescent population.

While efforts to strengthen healthcare systems are ongoing, our study highlights the need for improved HIV data reporting systems, as evidenced by the high rate of missing data in the dataset. Notably, the extent of missing data was particularly high during the COVID-19 pandemic, a trend similarly reported in multiple studies because of the pandemic’s widespread impact. For example, in Malawi, viral load testing coverage declined from 60% pre-COVID-19 to 40% during the pandemic [37]. This decline has been attributed to several factors, including lockdowns that restricted access to healthcare facilities and the reallocation of resources to address the pandemic. These measures led to the temporary closure of some health facilities and the suspension of viral load testing in others [38, 39, 40]. Additionally, adaptations to service delivery models during the pandemic likely influenced data completeness. Measures such as decentralizing ART refill centres to community outreach programs, extending ART refill durations to multi-month supplies, transitioning to remote service delivery, and altering the traditional face-to-face clinic appointment model were widely implemented in sub-Saharan Africa [41, 42]. These changes, while necessary, may have contributed to inconsistencies and gaps in data reporting.

The study also revealed delays in HIV diagnosis and ART initiation among adolescents, particularly given that sexual debut occurs at the age of 15 years for at least 3% of adolescents in Eswatini, suggesting that HIV transmission in these cases is most likely vertical [32]. This is despite the introduction and implementation of prevention of mother-to-child transmission (PMTCT) programs and the WHO universal test-and-treat guidelines in the country [23]. Delayed HIV diagnosis is associated with increased chances of unsuppressed viral load due to elevated barriers to ART adherence associated with delayed status disclosure. Delayed status disclosure has been associated with delayed status acceptance which is linked to self-stigma, anger, and loss of hope among adolescents [17]. Adolescents who were initiated on ART at paediatric level are most likely to adhere to the medication when compared to those diagnosed and initiated on ART later in life [43]. This concept has been explained by Toromo et al. (2022) to result from the status acceptance process which begins with anger, followed by denial then depression before acceptance [17, 44]. During the status acceptance process, retention in care tends to be low, with high rates of loss to follow-up and poor ART adherence, which are associated with unsuppressed viral loads and AIDS-related deaths [17]. There is therefore need for interventions aimed at scaling up early HIV testing and diagnosis among children born to HIV positive mothers, continuous support to ensure early ART initiation after diagnosis.

The study also uncovered that VLS was higher among older adolescents (89.3%) compared to younger adolescents (86.9%), although this difference was not statistically significant. However, contrasting findings have been reported in studies conducted in South Africa, Rwanda, and Uganda, where VLS was found to be lower among older adolescents [45, 46, 47]. The VLS differences between the two age groups have been attributed to various barriers, including reduced support, perceived stigma, and forgetfulness [48, 49]. These barriers contribute to sub-optimal adherence to ART, which, in turn, is associated with non-viral load suppression [45, 49].

Additionally, our study found no statistically significant difference in VLS rates between females (88.9%) and males (87.8%). This contrasts with findings from other studies, which have reported lower VLS rates among males, often attributed to reluctance to access health services [50]. Despite this observed trend in other research, our study did not identify a sex-based disparity in VLS rates among adolescents living with HIV.

Furthermore, although previous studies have reported an association between regimen and ART duration and VLS, our study found no statistically significant association between either regimen or ART duration and VLS [51]. Contrary to findings from previous studies reporting a negative association between second-line regimen and VLS, as well as an association between increasing ART duration and a four-fold increase in the likelihood of non-suppressed viral load, our study did not identify such associations [45, 47]. This observed decrease in VLS is often associated with factors such as pill fatigue, forgetfulness, and the age of adolescents [6, 48, 49].

In this study, only residence in the Shiselweni region and main-stream model of ART care were associated with VLS in the bivariable analysis. These were both negatively associated with VLS. A systematic review of studies reporting intervention to improve ART outcomes among adolescents reported that differentiated service delivery models aimed at support such as peer-led, community support groups and family based economic empowerment were positively associated with VLS when compared to main-stream or standard of care service delivery model [12]. However, in our study, the multivariable analysis revealed that only residence in the Shiselweni region was statistically associated with VLS.

The Shiselweni region, situated in the southern part of the country, is predominantly characterized as a rural community with limited health service infrastructure [52]. Moreover, studies have indicated that the implementation of the universal test and treat (UTT) HIV program in this region has resulted in low retention in care [52, 53]. This is attributed to factors such as non-acceptance of one’s HIV positive status upon diagnosis, transport costs and the distance to health facilities [52, 53, 54, 55]. It is noteworthy that low retention in care is strongly associated with suboptimal adherence to ART and subsequent non-suppressed viral load [3]. This is further supported by findings from a study conducted in South Africa, where distance to health facilities was identified as a significant barrier to ART adherence and, consequently, viral load suppression due to low retention in care [56]. Nevertheless, the introduction of community ART distribution and community ART outreach in Eswatini’s hard-to-reach communities in the Shiselweni region has shown positive effects on retention to care among people living with HIV aged 16 years and above [57].

The national data further highlights that the Shiselweni region bears the highest HIV prevalence rate at 26.5%, surpassing other regions [8]. However, intriguingly, our study findings contrast with this trend, revealing that Shiselweni had the lowest proportion of adolescents living with HIV (ALHIV) compared to the other region**s.** While the national findings suggest no direct association between the high HIV prevalence and viral load suppression (VLS) in the region, our study uncovers an association between the region and VLS among ALHIV [10]. Studies have revealed that unsuppressed viral load is associated with delayed HIV testing, lower CD4-count associated with HIV symptoms, delayed ART initiation and multiple missed or late appointments [45, 58, 59]. These were further associated with limited health services which has been reported in the Shiselweni region [52]. These results suggest that interventions aimed at early HIV testing should be scaled up among adolescents in the Shiselweni region, while also improving ART adherence should not solely rely on HIV prevalence rate and geographical location. This is consistent with the findings of studies emphasizing the need for tailored and context-specific approaches to ART adherence interventions, irrespective of regional HIV prevalence rates [56, 60, 61].

### Study strength and limitation

The strength of our study is that the results demonstrate the current VLS level and key associated factors (demographic and social, HIV-related, and facility-related) among ALHIV aged 10 to 19 years, a population group normally reported with young adults and paediatrics. Secondly, since the study used the national dataset, the study results can extrapolate the current VLS situation in Eswatini specifically among ALHIV. Additionally, since secondary data was used, recall and reporting bias were eliminated. Also, the data collection process was informed by professionalism and expertise, and not associated with research thus was not subject to interview bias. Lastly, being the only study conducted among adolescents within this age group, our study can contribute to informing policies, plans, and service delivery strategies for preventing and controlling poor ART adherence among ALHIV in Eswatini.

Nonetheless, some limitations should be considered when interpreting the findings of this study. Firstly, the study used secondary data for analysis that were not intentionally collected for this study and therefore limited the study in evaluating other predictors of VLS which were not covered in the records. Secondly, being a secondary dataset, the data was prone to missing variables due to incomplete documentation. Thirdly, the nature of the study design being cross-sectional also was a limitation as changes over time in terms of viral load suppression could not be ascertained. Lastly, our inclusion criteria permitted the inclusion of records with only a single RNA result, specifically the most recent result conducted six months prior to data abstraction. Unfortunately, this approach excluded records with earlier RNA test results, particularly because the National HIV Treatment Guidelines recommend RNA testing once a year for individuals with undetectable viral load in two consecutive results [24]. As a result, records with RNA results conducted more than six months before data abstraction were missed resulting in the generalizability of study the findings. However, we are confident that the available data provide true estimation as it was captured by qualified health professionals.

## Conclusions

In conclusion, our study reveals that VLS remains low among ALHIV in Eswatini, despite significant progress toward achieving the 95-95-95 UNAIDS 2030 target in the general population. These findings underscore the urgent need for innovative strategies to enhance early ART initiation and provide intensive follow-up and support for ALHIV, particularly in the Shiselweni region.

## Electronic supplementary material

Below is the link to the electronic supplementary material.

Supplementary Material 1

## Data Availability

All data supporting the findings of this study are available within the paper.

## Abbreviations

AIDS: Acquired immunodeficiency syndrome
HIV: Human immunodeficiency virus
ALHIV: Adolescents living with HIV
ART: Antiretroviral therapy
VLS: Viral load suppression
PLHIV: People living with HIV
UNAIDS: The joint united nations programme on HIV/AIDS
UTT: Universal test and treat
SSA: Sub-saharan africa
CMIS: Client management information system
LHW: Lay health worker
CD4: Cluster of differentiation 4
NRTIs: Nucleoside reverse-transcriptase inhibitors
NNRTIs: Non-nucleoside reverse-transcriptase inhibitors
NGO: Non-government organization

## References

[1] UNAIDS Global HIV & AIDS statistics — Fact sheet| UNAIDS. 2023. Available from: https://www.unaids.org/en/resources/fact-sheet

[2] WHO Global HIV Programme. 2024. Available from: https://www.who.int/teams/global-hiv-hepatitis-and-stis-programmes/hiv/treatment/service-delivery-adherence-retention

[3] Hine P, Smith R, Eshun-Wilson I, Orrell C, Cohen K, Leeflang MMG, et al. Measures of antiretroviral adherence for detecting viral non-suppression in people living with HIV. Cochrane Database Syst Rev. 2018;2018:7. 10.1002/14651858.CD013080.pub2 PMC9309033 35871531

[4] UNICEF. HIV Statistics - Global and Regional Trends - UNICEF DATA. 2023. Available from: https://data.unicef.org/topic/hivaids/global-regional-trends/

[5] Vreeman RC, Rakhmanina NY, Nyandiko WM, Puthanakit T, Kantor R. Are we there yet? 40 years of successes and challenges for children and adolescents living with HIV. J Int AIDS Soc. 2021;24(6):e25759. Available from: https://onlinelibrary.wiley.com/doi/full/10.1002/jia2.25759 10.1002/jia2.25759 PMC8183166 34097352

[6] Hlophe LD, Jacques L, Tamuzi, Constance S, Shumba PSN. Barriers and facilitators to anti-retroviral therapy adherence among adolescents aged 10 to 19 years living with HIV in sub-Saharan Africa: A mixed-methods systematic review and meta-analysis. PLoS One. 2023;18(5):e0276411. Available from: 10.1371/journal.pone.0276411 10.1371/journal.pone.0276411 PMC10194875 37200399

[7] Justman J, Reed JB, Bicego G, Donnell D, Li K, Bock N, et al. Swaziland HIV incidence measurement survey (SHIMS): a prospective National cohort study. Lancet HIV. 2017;4(2):e83–92. 27863998 10.1016/S2352-3018(16)30190-4 PMC5291824

[8] SHIMS2. Swaziland H Incidence Measurement Survey 2: a Population-Based HIV Impact Assessment. 2017;1–4. Available from: http://phia.icap.columbia.edu/wp-content/uploads/2017/11/Swaziland_new.v8.pdf

[9] WHO. Eswatini achieves the 95-95-95 HIV treatment target - a decade ahead of 2030 goal| WHO| Regional Office for Africa. 2023. Available from: https://www.afro.who.int/countries/eswatini/news/eswatini-achieves-95-95-95-hiv-treatment-target-decade-ahead-2030-goal

[10] PEPFAR. Eswatini surpasses UNAIDS Fast-track Targets for treatment and viral suppression - United States Department of State. 2022. Available from: https://www.state.gov/eswatini-surpasses-unaids-fast-track-targets-for-treatment-and-viral-suppression/

[11] Nsibandze BS, Downing C, Poggenpoel M, Myburgh CPH. I have been rejected so many times experiences of female adolescents living with HIV in rural Manzini, Eswatini: A case study. Int J Afr Nurs Sci. 2021;14:100307.

[12] Munyayi FK, van Wyk B, Mayman Y. Interventions to improve treatment outcomes among adolescents on antiretroviral therapy with unsuppressed viral loads: A Systematic Review. Int J Environ Res Public Health. 2022;19(7). Available from: 10.3390/ijerph19073940 10.3390/ijerph19073940 PMC8997420 35409621

[13] Wakooko P, Gavamukulya Y, Wandabwa JN. Viral load suppression and associated factors among HIV patients on antiretroviral treatment in Bulambuli district, Eastern Uganda: A retrospective cohort study. Infect Dis (Auckl). 2020;13:1178633720970632. 33223836 10.1177/1178633720970632 PMC7656881

[14] Bermudez LG, Ssewamala FM, Neilands TB, Lu L, Jennings L, Nakigozi G et al. Does economic strengthening improve viral suppression among adolescents living with HIV? Results from a cluster randomized trial in Uganda. AIDS Behav 2018 2211. 2018;22(11):3763–72. Available from: 10.1007/s10461-018-2173-7https://link.springer.com/article/10.1007/s10461-018-2173-7 PMC6204092 29846836

[15] Nachega JB, Hislop M, Nguyen H, Dowdy DW, Chaisson RE, Regensberg L, et al. Antiretroviral therapy adherence, virologic and Immunologic outcomes in adolescents compared with adults in Southern Africa. JAIDS J Acquir Immune Defic Syndr. 2009;51(1):65–71. 19282780 10.1097/QAI.0b013e318199072e PMC2674125

[16] Bongfen MC, Torpey K, Ganle J, Ankomah A. Level of adherence and associated factors among HIV-positive adolescents on antiretroviral therapy in Cameroon. Afr J AIDS Res. 2020;19(4):269–75. 33337976 10.2989/16085906.2020.1833055

[17] Toromo JJ, Apondi E, Nyandiko WM, Omollo M, Bakari S, Aluoch J et al. I have never talked to anyone to free my mind– challenges surrounding status disclosure to adolescents contribute to their disengagement from HIV care: a qualitative study in western Kenya. BMC Public Health. 2022;22(1):1–10. Available from: https://link.springer.com/articles/10.1186/s12889-022-13519-9 10.1186/s12889-022-13519-9 PMC9167528 35658924

[18] Apondi E, Wachira J, Ayikukwei R, Kafu C, Onyango J, Omollo M, et al. Barriers to ART adherence among school students living with HIV in Kenya. Afr J AIDS Res. 2021;20(3):232–7. 34635018 10.2989/16085906.2021.1979606

[19] Gill MM, Ndimbii JN, Otieno-Masaba R, Ouma M, Jabuto S, Ochanda B. Adherence challenges and opportunities for optimizing care through enhanced adherence counseling for adolescents with suspected HIV treatment failure in Kenya. BMC Health Serv Res. 2022;22(1):1–11. Available from: https://link.springer.com/articles/10.1186/s12913-022-08373-9 10.1186/s12913-022-08373-9 PMC9336023 35906574

[20] Silvestre E. Measure Evaluation. Implementing Swaziland’s Client Management Information System: Stakeholder’s views of the process and recommendations to improve it. 2017;1-42. Available from: https://www.measureevaluation.org/resources/publications/tr-17-226.html

[21] Eswatini Ministry of Health. Eswatini Integrated HIV Management Guidelines. 2018. Available from: http://swaziaidsprogram.org/wp-content/uploads/2021/07/2018-Integrated-HIV-Management-Guidelines_final-1.pdf

[22] Swaziland National AIDS Programme. National facility teen clubs. Eswatini Ministry of Health. 2023.

[23] Eswatini Ministry of Health. Guidelines for Differentiated Service Delivery. Mbabane. 2022. Available from: https://cquin.icap.columbia.edu/wp-content/uploads/2022/07/Final_Policy-Guidelines-for-Eswatini-Differentiated-Service-Delivery.pdf

[24] Eswatini Ministry of Health. Amendment to the Eswatini Integrated HIV Management Guidelines. 2024; Available from: http://swaziaidsprogram.org/wp-content/uploads/2024/08/Eswatini-Integrated-HIV-Management-Guidelines-2.pdf

[25] Dlamini-Simelane T, Moyer E. Task shifting or shifting care practices? The impact of task shifting on patients’ experiences and health care arrangements in Swaziland. BMC Health Serv Res. 2017;17(1). 10.1186/s12913-016-1960-y PMC5223454 28069047

[26] Ahmed CV, Weissinger G, Teitelman A, Sabelo Dlamini N, Patience Dlamini N, Cebsile Dlamini T, et al. Expert client service delivery practices among adolescents living with HIV in Eswatini: A thematic analysis. Child Youth Serv Rev. 2022;132:106309.

[27] Eswatini Ministry of Health. Service Availability and Readiness Assessment. 2017. Available from: https://www.hst.org.za/publications/NonHSTPublications/Eswatini SARA March (05 04 2019).pdf.

[28] Magagula SV, A case study of the Swaziland Essential Health Care Package. 2017. Available from: https://www.equinetafrica.org/sites/default/files/uploads/documents/Swaziland EHB case study rep final2017pv.pdf.

[29] Dean AG, Sullivan KMSM. 2013. OpenEpi: Open Source Epidemiologic Statistics for Public Health, Version. Available from: https://www.openepi.com

[30] CDC. Centers for disease control and prevention. 2019. Technical Notes| Volume 26 Number 2| HIV Surveillance| Reports| Resource Library| HIV/AIDS| CDC. Available from: https://www.cdc.gov/hiv/library/reports/hiv-surveillance/vol-26-no-2/content/technical-notes.html

[31] StataCorp TX. 2019. Available from: https://www.stata.com/support/faqs/resources/citing-software-documentation-faqs/

[32] Eswatini Ministry of Health. Eswatini Population-based HIV Impact Assessment 3 2021 (SHIMS3 2021): Final Report. 2023. Available from: https://phia.icap.columbia.edu/wp-content/uploads/2023/12/241123_SHIMS_ENG_RR3_Final-1.pdf

[33] Abuonji EA, Aduda DO, Marete IK, Ochung’ AJ, Okanda IA, Owili PO et al. Household- based factors associated with viral load suppression among adolescents living with HIV in Western Kenya. Afr J Health Sci. 2023;36(5):547–56. Available from: https://www.ajol.info/index.php/ajhs/article/view/274565

[34] Desta AA, Woldearegay TW, Futwi N, Gebrehiwot GT, Gebru GG, Berhe AA, et al. HIV virological non-suppression and factors associated with non-suppression among adolescents and adults on antiretroviral therapy in Northern Ethiopia: a retrospective study. BMC Infect Dis. 2020;20(1):1–10. 10.1186/s12879-019-4732-6 PMC6941313 31898535

[35] Crowley T, van der Merwe A, Kidd M, Skinner D. Adolescent human immunodeficiency virus self-management: associations with treatment adherence, viral suppression, sexual risk behaviours and health-related quality of life. South Afr J HIV Med. 2020;21(1):1054. 32391177 10.4102/sajhivmed.v21i1.1054 PMC7203195

[36] Gebreeyesus F, Mitku D, Tarekegn T, Temere B, Terefe T, Belete A et al. Levels of adherence and associated factors among children on ART over time in Northwest, Ethiopia: Evidence from a multicenter follow- up study. 2021. Available from: 10.2147/HIV.S323090 10.2147/HIV.S323090 PMC8380805 34434060

[37] Kalua T, Egger M, Jahn A, Chimpandule T, Kolola R, Anderegg N. HIV suppression was maintained during the COVID-19 pandemic in Malawi: a program-level cohort study. J Clin Epidemiol. 2022;150:116–25. Available from: https://linkinghub.elsevier.com/retrieve/pii/S0895435622001706 10.1016/j.jclinepi.2022.06.019 PMC9249434 35788400

[38] Okegbe T, Williams J, Plourde KF, Oliver K, Ddamulira B, Caparrelli K. Impact of COVID-19 on HIV adolescent programming in 16 countries with USAID- supported PEPFAR programs. J Acquir Immune Defic Syndr. 2023;93(4):261–71. Available from: https://journals.lww.com/jaids/fulltext/2023/08010/impact_of_covid_19_on_hiv_adolescent_programming.1.aspx 10.1097/QAI.0000000000003201 PMC10287048 36989134

[39] Lowenthal ED, DeLong SM, Zanoni B, Njuguna I, Beima-Sofie K, Dow D et al. Impact of COVID-19 on adolescent HIV prevention and treatment research in the AHISA Network. AIDS Behav. 2022;1:1–11. Available from: 10.1007/s10461-022-03811-5https://link.springer.com/article/10.1007/s10461-022-03811-5 PMC9466311 36094636

[40] Ahmed CV, Brooks MJ, DeLong SM, Zanoni BC, Njuguna I, Beima-Sofie K et al. Impact of COVID-19 on adolescent HIV prevention and treatment services in the AHISA Network. AIDS Behav. 2023;27(1):84–93. Available from: 10.1007/s10461-022-03959-0https://link.springer.com/article/10.1007/s10461-022-03959-0 PMC9792928 36574183

[41] Bailey LE, Siberry GK, Agaba P, Douglas M, Clinkscales JR, Godfrey C. The impact of COVID-19 on multi-month dispensing (MMD) policies for antiretroviral therapy (ART) and MMD uptake in 21 PEPFAR-supported countries: a multi-country analysis. J Int AIDS Soc. 2021;24(S6):e25794. Available from: https://onlinelibrary.wiley.com/doi/full/10.1002/jia2.25794 10.1002/jia2.25794 PMC8554217 34713578

[42] Ahmed CV, Brooks MJ, DeLong SM, Zanoni BC, Njuguna I, Beima-Sofie K et al. Impact of COVID-19 on adolescent HIV prevention and treatment services in the AHISA Network. AIDS Behav. 2022;1–10. Available from: 10.1007/s10461-022-03959-0https://link.springer.com/article/10.1007/s10461-022-03959-0 PMC9792928 36574183

[43] Munyayi FK, van Wyk BE. Determinants and rates of retention in HIV care among adolescents receiving antiretroviral therapy in Windhoek, Namibia: a baseline cohort analysis. BMC Public Health. 2023;23(1):1–12. Available from: https://link.springer.com/articles/10.1186/s12889-023-15356-w 10.1186/s12889-023-15356-w PMC9994767 36890540

[44] Enane LA, Apondi E, Omollo M, Toromo JJ, Bakari S, Aluoch J, et al. I just keep quiet about it and act as if everything is alright– The cascade from trauma to disengagement among adolescents living with HIV in Western Kenya. J Int AIDS Soc. 2021;24(4):1–11. 10.1002/jia2.25695 PMC8035676 33838007

[45] Okonji EF, van Wyk B, Mukumbang FC, Hughes GD. Determinants of viral suppression among adolescents on antiretroviral treatment in ehlanzeni district, South Africa: a cross-sectional analysis. AIDS Res Ther. 2021;18(1):66. 34627300 10.1186/s12981-021-00391-7 PMC8501534

[46] Mutwa PR, Boer KR, Asiimwe-Kateera B, Tuyishimire D, Muganga N, Lange JMA, et al. Safety and effectiveness of combination antiretroviral therapy during the first year of treatment in HIV-1 infected Rwandan children: a prospective study. PLoS ONE. 2014;9(11):e111948. 25365302 10.1371/journal.pone.0111948 PMC4218827

[47] Maena J, Banke-Thomas A, Mukiza N, Kuteesa CN, Kakumba RM, Kataike H, et al. Determinants of viral load non-suppression among adolescents in Mbale district, Eastern rural Uganda. AIDS Res Ther. 2021;18(1):91. 34863196 10.1186/s12981-021-00408-1 PMC8642852

[48] Ankrah DNAA, Koster ES, Mantel-Teeuwisse AK, Arhinful DK, Agyepong IA, Lartey M. Facilitators and barriers to antiretroviral therapy adherence among adolescents in Ghana. Patient Prefer Adherence. 2016;10:329–37. 27042024 10.2147/PPA.S96691 PMC4801129

[49] Adejumo OA, Malee KM, Ryscavage P, Hunter SJ, Taiwo BO. Contemporary issues on the epidemiology and antiretroviral adherence of HIV-infected adolescents in sub-Saharan Africa: A narrative review. J Int AIDS Soc. 2015;18(1). 10.7448/IAS.18.1.20049 PMC4575412 26385853

[50] Maena J, Banke-Thomas A, Mukiza N, Kuteesa CN, Kakumba RM, Kataike H et al. Determinants of viral load non-suppression among adolescents in Mbale District, Eastern Rural Uganda. AIDS Res Ther. 2021;18(1):1–9. Available from: https://link.springer.com/articles/10.1186/s12981-021-00408-1 10.1186/s12981-021-00408-1 PMC8642852 34863196

[51] Natukunda J, Kirabira P, Ong KIC, Shibanuma A, Jimba M. Virologic failure in HIV-positive adolescents with perfect adherence in Uganda: a cross-sectional study. Trop Med Health. 2019;47(1):1–10. 30679930 10.1186/s41182-019-0135-z PMC6337787

[52] Jobanputra K, Parker LA, Azih C, Okello V, Maphalala G, Kershberger B, et al. Factors associated with virological failure and suppression after enhanced adherence counselling, in children, adolescents and adults on antiretroviral therapy for HIV in Swaziland. PLoS ONE. 2015;10(2):e0116144. 25695494 10.1371/journal.pone.0116144 PMC4335028

[53] Horter S, Thabede Z, Dlamini V, Bernays S, Stringer B, Mazibuko S et al. Life is so easy on ART, once you accept it: Acceptance, denial and linkage to HIV care in Shiselweni, Swaziland. Soc Sci Med. 2017;176:52–9. Available from: 10.1016/j.socscimed.2017.01.006 10.1016/j.socscimed.2017.01.006 28129547

[54] Kerschberger B, Schomaker M, Jobanputra K, Kabore SM, Teck R, Mabhena E et al. HIV programmatic outcomes following implementation of the Treat-All policy in a public sector setting in Eswatini: a prospective cohort study. J Int AIDS Soc. 2020;23(3). Available from: https://pubmed.ncbi.nlm.nih.gov/32128964/10.1002/jia2.25458 PMC7054447 32128964

[55] Barbara Sibbald. Responding to Swaziland’s dual epidemic. CMAJ. 2013;185(1):E13–4. Available from: https://www.cmaj.ca/content/cmaj/185/1/E13.full.pdf 10.1503/cmaj.109-4334 PMC3537796 23184847

[56] Cluver L, Pantelic M, Toska E, Orkin M, Casale M, Bungane N, et al. STACKing the odds for adolescent survival: health service factors associated with full retention in care and adherence amongst adolescents living with HIV in South Africa. J Int AIDS Soc. 2018;21(9):e25176. 30240121 10.1002/jia2.25176 PMC6149366

[57] Pasipamire L, Nesbitt RC, Ndlovu S, Sibanda G, Mamba S, Lukhele N et al. Retention on ART and predictors of disengagement from care in several alternative community-centred ART refill models in rural Swaziland. J Int AIDS Soc. 2018;21(9):e25183. Available from: https://onlinelibrary.wiley.com/doi/full/10.1002/jia2.25183 10.1002/jia2.25183 PMC6141897 30225946

[58] Mwangi A, van Wyk B. Factors Associated with viral suppression among adolescents on antiretroviral therapy in Homa Bay County, Kenya: A retrospective cross- sectional study. HIV/AIDS - Res Palliat Care. 2021;13:1111–8. Available from: 10.2147/HIV.S345731 10.2147/HIV.S345731 PMC8713714 34992469

[59] Mchomvu RD, Hussein AK, Matee M. Determinants of viral load non-suppression among HIV-positive children and adolescents attending care and treatment clinics in Tabora region, Tanzania. Bull Natl Res Cent 2022 461. 2022;46(1):1–9. Available from: https://link.springer.com/articles/10.1186/s42269-022-00961-3

[60] Fite RO. Association between adherence to Antiretroviral Therapy and place of residence among adult HIV infected patients in Ethiopia: A systematic review and meta-analysis. Madeddu G, editor. PLoS One. 2021;16(9):e0256948. Available from: 10.1371/journal.pone.0256948 10.1371/journal.pone.0256948 PMC8412366 34473774

[61] Ng’ambi WF, Estill J, Jahn A, Orel E, Chimpandule T, Nyirenda R et al. Factors associated with < scp > HIV viral suppression among children and adults receiving antiretroviral therapy in Malawi in 2021: Evidence from the Laboratory Management Information System. Trop Med Int Heal. 2022;27(7):639–46. Available from: https://onlinelibrary.wiley.com/doi/10.1111/tmi.13782 10.1111/tmi.13782 35622358

