# Supplementary material for: Determinants of viral load suppression among adolescents on antiretroviral therapy in Eswatini: a cross-sectional study
Source: BMC Infect Dis. 2025 Apr 10;25:493. doi: 10.1186/s12879-025-10872-z (PMC11983843; doi:10.1186/s12879-025-10872-z)
Supplement: Supplementary file 1 — Supplementary Material 1 [file 12879_2025_10872_MOESM1_ESM.docx]

**Supplementary table. Characteristics of abstracted records showing both excluded and included records**

| **Variable** | **Total Records** | **Excluded record (%)** | **Included records**  **(%)** |
| --- | --- | --- | --- |
| **Age group** | | | |
| 10-14 | 908 | 603 (66.4) | 305 (33.6) |
| 15-19 | 2512 | 1906 (75.9) | 606 (24.1) |
| Total | 3420 | 2509 (73.4) | 911 (26.6) |
| **Sex** | | | |
| Female | 2416 | 1865 (77.2) | 551 (22.8) |
| Male | 998 | 638 (63.9) | 360 (36.1) |
| Total | 3414 | 2503 (73.3) | 911 (26.7) |
| **Region** | | | |
| Hhohho | 891 | 536 (60.2) | 355 (39.8) |
| Lubombo | 757 | 573 (75.7) | 184 (24.3) |
| Manzini | 1247 | 1008 (80.8) | 239 (19.2) |
| Shiselweni | 525 | 392 (74.7) | 133 (25.3) |
| Total | 3420 | 2509 (73.4) | 911 (26.6) |
| **Facility ownership** | | | |
| Government | 2096 | 1455 (70.7) | 641 (30.6) |
| NGO | 593 | 470 (79.3) | 123 (20.7) |
| Faith-based | 730 | 583 (79.9) | 147 (20.1) |
| Total | 3419 | 2508 (73.4) | 911 (26.6) |
| **ART duration** | | | |
| <2 years | 1120 | 967 (86.3) | 153 (13.7) |
| ≥2years | 1639 | 881 (53.8) | 758 (46.2) |
| Total | 2759 | 1848 (70.0) | 911 (30.0) |
| **ART regimen** | | | |
| 1^st^ Line | 2114 | 1684 (79.7) | 430 (20.3) |
| 2^nd^ Line | 37 | 20 (54.1) | 17 (45.9) |
| Total | 2151 | 1704 (79.2) | 447 (20.8) |
| **WHO Clinical Stage** | | | |
| I-II | 134 | 53 (39.6) | 81 (60.4) |
| III-IV | 146 | 86 (58.9) | 60 (41.1) |
| Total | 280 | 139 (49.6) | 141 (50.4) |
| **Model of ART Care** | | | |
| Multi-Months | 168 | 102 (60.7) | 66 (39.3) |
| Mainstream | 1544 | 1111 (72.0) | 433 (28.0) |
| Teen Clubs | 563 | 225 (40.0) | 338 (60.0) |
| Fast Track | 46 | 29 (63.0) | 17 (37.0) |
| Total | 2321 | 1467 (63.2) | 854 (36.8) |
